# Dynamical analysis of scabies delayed epidemic model with second-order global stability

**DOI:** 10.1371/journal.pone.0319095

**Published:** 2025-04-21

**Authors:** Emad Fadhal, Ali Raza, Eugénio M. Rocha, Wafa F. Alfwzan, Muhammad Rafiq, Nauman Ahmed, Muhammad Bilal

**Affiliations:** 1 Department of Mathematics and Statistics, College of Science, King Faisal University, Al-Ahsa, Saudi Arabia; 2 Department of Physical Sciences, The University of Chenab, Gujrat, Pakistan; 3 Department of Computer Science and Mathematics, Lebanese American University, Beirut, Lebanon; 4 Center of Research and Development in Mathematics and Application, Department of Mathematics, University of Aveiro, Aveiro, Portugal; 5 Department of Mathematical Sciences, College of Science, Princess Nourah Bint Abdulrahman University, Riyadh, Saudi Arabia; 6 Department of Mathematics, Namal University, Mianwali, Pakistan; 7 Department of Mathematics and Statistics, The University of Lahore, Lahore, Pakistan; University of Jeddah, SAUDI ARABIA

## Abstract

Scabies is a highly transmitted skin disease that can affect people of all ages, especially children. According to the World Health Organization (WHO), South Asia and sub-Saharan Africa are the regions most affected. For the study of the dynamics of scabies in the population, the mathematical model is designed with delay differential equations (DDEs) for four subpopulations: unvaccinated individuals, vaccinated individuals, infected individuals, and recovered individuals. The fundamental properties of the model, such as positivity, boundedness, existence, and uniqueness, are proved. The equilibria, reproduction number, sensitivity analysis, and (Local and Global) stabilities for the second order are studied rigorously. The numerical simulations were performed to confirm the validity of their theoretical results. The study’s findings suggest delay-based modeling of scabies and the advanced stability analysis provides a better understanding of epidemic management and disease dynamics over time.

## Introduction

Scabies is a really contagious skin infestation caused by the Sarcoptes scabies mite that causes extreme itching and a rash. Scabies modeling is essential to understand the dynamic transmission, assess control strategies, and forecast general patterns of outbreaks. Efficient models are also important to advocate public health contesting its spread, particularly among; vulnerable and densely packed communities. There are some types of modeling have been made by other authors as follows: Bhunu et al. described the assessment of the effect of vaccination on controlling the spread of human Scabies [1]. Rashid et al. studied mathematical modeling to apply fractional-fractal derivatives of nonlinear Scabies in the dynamics [2]. Mhlanga et al. described how to control and analyze the cost-effectiveness of a scabies model with both direct and indirect transmissions [3]. Hindle et al. defined mathematical modeling of the behaviour of environmentally transmitted diseases as influenced by their spatial dynamics [4]. Browne et al. studied the transmission dynamics of scabies in different host species [5]. Niode et al. investigated mathematical modeling to study crusted Scabies, a disease often overlooked and affecting people in tropical regions [6]. Fantaye et al. noted mathematical modeling and analyzed its stability and how skin sores spread and influence people [7]. Launay et al. studied how the COVID-19 pandemic impacted the spread and dynamics of scabies infestations and head lice in the population of France [8]. Clark et al. considered how using mathematical modeling can assist in guiding the actions and plans outlined by the World Health Organization between 2021 and 2030 to address neglected tropical diseases [9]. Marks et al. optimized mathematical modeling for diagnosing Scabies to help avoid Scabies–the development of two target product profiles [10]. Tellioglu et al. studied mathematical modeling to test different sampling techniques to see which ones are most effective in estimating the prevalence of Scabies [11]. Rinaldi et al. conducted a systematic review that assessed the use of mathematical modeling in evaluating the administration of mass drugs for controlling Scabies in areas where the disease is spread [12]. Tellioglu et al. conducted mathematical modeling to determine how well mass drug administration techniques reduce the burden of Scabies in Monrovia, Liberia [13]. Winarni et al. described the mathematical model called “Epidemic SEITS” that includes a linear occurrence in the spread of scabies disease [14]. Marks et al. gained valuable knowledge from mathematical modeling to understand how the proposed WHO goals for Scabies in 2030 can be achieved [15]. Linden et al. conducted a study that reviewed different scabies models of transmission and data to determine the various cost-effective interventions for managing the disease [16]. Lydeamore et al. studied a brainstormed way to calculate the age when people in five distant communities in northern Australia likely get their scabies infection and first skin sores [17]. Engelman et al. explained how mathematical modeling can help control Scabies and identified the most critical areas for further research and action to tackle this human disease [18]. Romani et al. observed how mathematical modeling holds the worldwide generality of Scabies and gives a systematic review of Scabies [19]. Whittle et al. studied, with the help of mathematical modeling, clinical and epidemiological revealed Scabies, pyoderma, and nephritis in Zaria and Nigeria [20]. Ahmed et al. studied the mathematical modeling to understand how Scabies, an infectious disease caused by mites, identify ways to prevent or manage it more effectively in children in Saudia Arabic [21].

Scabies present a significant challenge in developing regions such as Asia, Africa, and Europe. While vaccination efforts have made strides in developed countries, even the United States, a significant contributor, has faced challenges with nefarious purposes. This model is important in that it offers a way of improving our understanding of scabies transmission dynamics through realistic features such as the presence of delays in the infectious period which may not be captured in simpler models.

The paper’s structure unfolds: The introduction section explains about scabies-like diseases. The model formulation section delves into the delay differential equations (DDEs) and mathematical analysis. The ongoing sections address the local and global stability of the model. The sensitivity of the parameters of the model was discussed. At the end of sections, numerical simulations along with their results and concluding remarks summarizing the study’s key findings.

### Model formulation

Before going to the model formulation, the idea of delay incorporation into the model is important to understand. These delays have real consequences in the real world: they capture such things as the incubation period between infection and onset of symptoms and the time delay in getting treatment. By doing this, the model captures the immediacy of the response to an outbreak, facilitating further transmission of disease or place changes in stability. By introducing delays, the study presents a more realistic dynamic and is therefore more relevant for developing effective intervention programs. The population N(t) is the sum of the following classes: unvaccinated susceptible $S_{u}(t)$, vaccinated susceptible $S_{V}(t,$infected I(t), and recovered R(t). The parameters of the model described as “Λ” and “α” are rates of birth and vaccination of unvaccinated susceptible class respectively. “ρ” is rate of vaccine wanes for vaccinated susceptible and move back into unvaccinated susceptible class. “λ” is a rate at which unvaccinated susceptible are infected with scabies where $\lambda =\frac{\beta I}{N}$ with “β” being the product of the probability of getting infected per contact with an infected case. Susceptible individuals interact with infected and carrier classes at any given time (t−τ). An artificial delay term, represented by $e^{-\mu \tau },\tau \geq 0$ (a decay term), regulates the epidemic. “γ” is a rate treatment of infected individuals. “μ” is a rate of natural death of all subclasses. Also, disease death rate is ignored because scabies do not kill people. “δλ”with 0<δ<1 is a reinfection rate of vaccinated susceptible individuals and “σλ”, σ∈(0,1) is a reinfection rate of recovered individuals.

**Fig 1:**
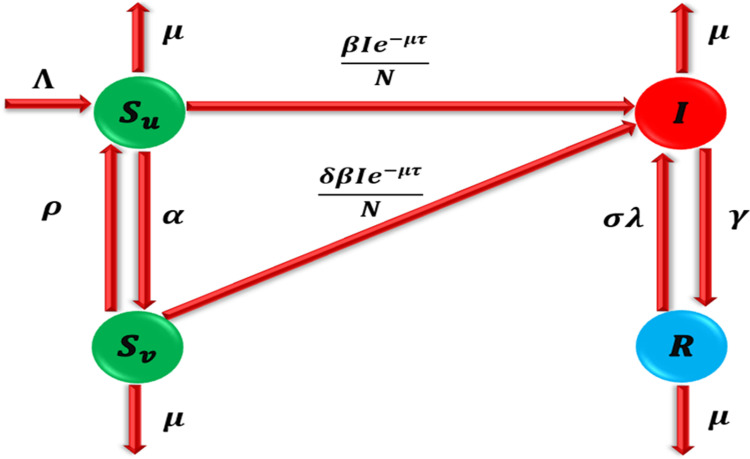
Model diagram.


Utilizing the outlined assumptions, the continuous model is formulated using the law of mass action. Fig 1 presents the transmission dynamics of Scabies-type diseases expressed through nonlinear delay differential equations (DDEs) in the following manner:

$$\frac{dS_{u}(t)}{dt}=\Lambda +\rho S_{v}(t)-\frac{\beta I(t-\tau )}{N}S_{u}(t-\tau )e^{-\mu \tau }-(\mu +\alpha )S_{u}(t),t\geq 0.$$ (1)

$$\frac{dS_{v}(t)}{dt}=\alpha S_{u}(t)-\delta \frac{\beta I(t-\tau )}{N}S_{v}{(t-\tau )e}^{-\mu \tau }-(\mu +\rho )S_{v}(t),t\geq 0.$$ (2)

$$\frac{dI(t)}{dt}=\frac{\beta I(t-\tau )}{N}S_{u}{(t-\tau )e}^{-\mu \tau }+\delta \frac{\beta I(t-\tau )}{N}S_{v}(t-\tau )e^{-\mu \tau }+\sigma \lambda R(t)-(\mu +\gamma )I(t),t\geq 0.$$ (3)

$$\frac{dR(t)}{dt}=\gamma I(t)-\sigma \lambda R(t)-\mu R(t),t\geq 0.$$ (4)

With non-negative initial conditions $S_{u}(0)\geq 0,S_{V}(0)\geq 0,I(0)\geq 0,R(0)\geq 0$ and t≥0,τ<t.

## Properties

To maintain a meaningful analysis of the model, it is imperative that all variables, namely $S_{u}(t),S_{v}(t),I(t),R(t)$, remain non-negative. As a result, the outcomes observed during the study of the model at any given time t (where t≥0 and τ<t) must fall within a feasible region.


$$ℬ=\left\{\begin{array}{c} \left(S_{u},S_{v},I,R\right)\in {\mathbb{R}}_{+}^{4}:N(t)\leq \frac{\Lambda }{\mu },\\ S_{u}(0)\geq 0,S_{V}(0)\geq 0,I(0)\geq 0,R(0)\geq 0 \end{array}\right\}.$$

**Theorem 1 (positivity):** The solutions ($S_{u},S_{v},I,R\in {\mathbb{R}}_{+}^{4}$ of the system (1–4) are positive at any time t≥0,τ<t with given nonnegative initial conditions.

**Proof:** Let us start from the class $S_{v}(t)\geq S_{v}(0)e^{-\left(\mu +\rho +\frac{\beta }{N}\delta e^{-\mu \tau }I_{\infty }\right)t}\geq 0,$

For the functionI(t), the following inequalities hold:


$$I(t)\geq I(0)e^{-(\mu +\gamma )t}\geq 0,$$

And


$$R(t)\geq R(0)e^{-\mu t}\geq 0.$$

We shall define the norm


$${⋋}_{\infty }=\sup_{t\in D_{⋋}}\left|⋋(t)\right|$$

Where $D_{⋋}$ is the domain of ⋋. Using the above norm, the inequalities for the function $S_{u}$(t) are defined.


$$\frac{dS_{u}}{dt}=\Lambda +\rho S_{v}-\frac{\beta I}{N}S_{u}e^{-\mu \tau }-(\mu +\alpha )S_{u.}$$

$$\frac{dS_{u}}{dt}\geq -\left(\mu +\alpha +\frac{\beta }{N}|I|e^{-\mu \tau }\right)S_{u},$$

$$\frac{dS_{u}}{dt}\geq -\left(\mu +\alpha +\frac{\beta }{N}e^{-\mu \tau }\sup_{t\in D_{⋋}}|I|\right)S_{u},$$

$$\frac{dS_{u}}{dt}\geq -\left(\mu +\alpha +\frac{\beta }{N}e^{-\mu \tau }I_{\infty }\right)S_{u},$$

$$S_{u}(t)\geq S_{u}(0)e^{-(\mu +\alpha +\frac{\beta }{N}e^{-\mu \tau }I_{\infty })t}\geq 0,asdesired.$$

**Theorem 2 (boundedness):** The solutions ($S_{u},S_{v},I,R\in {\mathbb{R}}_{+}^{4}$of the system (1–4) are bounded and lie in the feasible region ℬ if$\lim_{t⟶\infty }SupN(t)\leq \frac{\Lambda }{\mu }.$

**Proof**: Let us consider a population function for the particular model as:


$$N(t)=S_{u}(t)+S_{v}(t)+I(t)+R(t).$$

$$\frac{dN}{dt}=\Lambda -\mu \left(S_{u}+S_{v}+I+R\right).$$

$$\frac{dN}{dt}=\Lambda -\mu N.$$

$$N(t)=N(0)e^{-\mu t}+\frac{\Lambda }{\mu },\lim_{t⟶\infty }SupN(t)\leq \frac{\Lambda }{\mu },asdesired.$$

We shall define the norm ${⋋}_{\infty }=\sup_{t\in D_{⋋}}\left|⋋(t)\right|$ and consider the Banach space [22]. We present here the existence and uniqueness of the solution piece wisely.

We need to verify growth and Lipschitz condition properties to obtain such results. Let us consider the four positive constants $M_{1},M_{2},M_{3}andM_{4}<\infty ,suchthat{S_{u}}_{\infty }<M_{1},{S_{v}}_{\infty }<M_{2},I_{\infty }<M_{3}andR_{\infty }<M_{4}$. We have.


$$\left\{ \begin{array}{c} {S_{u}}^{\prime }=f_{1}(S_{u},S_{v},I,R,t)\\ {S_{v}}^{\prime }=f_{2}(S_{u},S_{v},I,R,t)\\ I^{\prime }=f_{3}(S_{u},S_{v},I,R,t)\\ R^{\prime }=f_{4}(S_{u},S_{v},I,R,t) \end{array},t\geq 0\right.$$

∀i=1,2,3,4.We first verify that


$${\left|f_{i}(S_{u},t)\right|}^{2}<k_{i}\left(1+{\left|{S_{u}}_{i}\right|}^{2}\right),$$

$${\left|f_{i}\left({S_{u}}^{1},t\right)-f_{i}\left({S_{u}}^{2},t\right)\right|}^{2}<\overset{―}{k_{i}}{\left|{S_{u}}^{1}-{S_{u}}^{2}\right|}^{2}.$$

For proof, we consider the function $f_{1}(S_{u},S_{v},I,R,t)$ and the following estimations hold ${\left|f_{1}(S_{u},S_{v},I,R,t)\right|}^{2}={\left|\Lambda +\rho S_{v}-\frac{\beta I}{N}S_{u}e^{-\mu \tau }-(\mu +\alpha )S_{u}\right|}^{2},$


$${\left|f_{1}(S_{u},S_{v},I,R,t)\right|}^{2}\leq 4|\Lambda {|}^{2}+4{\left|\rho S_{v}\right|}^{2}+4{\left|\frac{\beta I}{N}S_{u}e^{-\mu \tau }\right|}^{2}+4{\left|(\mu +\alpha )S_{u}\right|}^{2},$$

$${\left|f_{1}(S_{u},S_{v},I,R,t)\right|}^{2}\leq 4\left(|\Lambda {|}^{2}+\sup_{t\in D_{⋋}}{\left|\rho S_{v}\right|}^{2}+\sup_{t\in D_{⋋}}{\left|\frac{\beta I}{N}S_{u}e^{-\mu \tau }\right|}^{2}+\sup_{t\in D_{⋋}}{\left|(\mu +\alpha )S_{u}\right|}^{2}\right),$$

$${\left|f_{1}(S_{u},S_{v},I,R,t)\right|}^{2}\leq 4\left(|\Lambda {|}^{2}+|\rho {|}^{2}{S_{v}}_{\infty }+{\left|\frac{\beta }{N}\right|}^{2}I_{\infty }{S_{u}}_{\infty }e^{-\mu \tau }+|\mu +\alpha {|}^{2}{S_{u}}_{\infty }\right),$$

$${\left|f_{1}(S_{u},S_{v},I,R,t)\right|}^{2}\leq 4\left(|\Lambda {|}^{2}+|\mu +\alpha {|}^{2}{S_{u}}_{\infty }\right)\left(1+\frac{{\left|\frac{\beta }{N}\right|}^{2}{e^{-\mu \tau }I}_{\infty }{S_{u}}_{\infty }+{{|\rho {|}^{2}S}_{v}}_{\infty }}{|\Lambda {|}^{2}+|\mu +\alpha {|}^{2}{S_{u}}_{\infty }}\right).$$

The condition $\frac{{\left|\frac{\beta }{N}\right|}^{2}{e^{-\mu \tau }I}_{\infty }{S_{u}}_{\infty }+{{|\rho {|}^{2}S}_{v}}_{\infty }}{|\Lambda {|}^{2}+|\mu +\alpha {|}^{2}{S_{u}}_{\infty }}<1$, implies


$${\left|f_{1}(S_{u},S_{v},I,R,t)\right|}^{2}<k_{1}\left(1+{\left|S_{u}\right|}^{2}\right).$$

By using the same methodology, we get


$${\left|f_{2}(S_{u},S_{v},I,R,t)\right|}^{2}={\left|\alpha S_{u}-\delta \frac{\beta I}{N}S_{v}e^{-\mu \tau }-(\mu +\rho )S_{v}\right|}^{2}$$

$${\left|f_{2}(S_{u},S_{v},I,R,t)\right|}^{2}\leq 3{\left|\alpha S_{u}\right|}^{2}+3{\left|\delta \frac{\beta I}{N}S_{v}e^{-\mu \tau }\right|}^{2}+3{\left|(\mu +\rho )S_{v}\right|}^{2},$$

$${\left|f_{2}(S_{u},S_{v},I,R,t)\right|}^{2}\leq 3(\sup_{t\in D_{⋋}}{\left|\alpha S_{u}\right|}^{2}+\sup_{t\in D_{⋋}}{\left|\delta \frac{\beta I}{N}S_{v}e^{-\mu \tau }\right|}^{2}+\sup_{t\in D_{⋋}}{\left|(\mu +\rho )S_{v}\right|}^{2}),$$

$${\left|f_{2}(S_{u},S_{v},I,R,t)\right|}^{2}\leq 3(|\alpha {|}^{2}{S_{u}}_{\infty }+{\left|\delta \frac{\beta }{N}\right|}^{2}I_{\infty }{S_{v}}_{\infty }e^{-\mu \tau }+|\mu +\rho {|}^{2}{S_{v}}_{\infty }),$$

$${\left|f_{2}(S_{u},S_{v},I,R,t)\right|}^{2}\leq 3(|\mu +\rho {|}^{2}{S_{v}}_{\infty }\left(1+\frac{|\alpha {|}^{2}{S_{u}}_{\infty }+{\left|\delta \frac{\beta }{N}\right|}^{2}I_{\infty }{S_{v}}_{\infty }e^{-\mu \tau }}{|\mu +\rho {|}^{2}{S_{v}}_{\infty }}\right)$$

Under the condition $\frac{|\alpha {|}^{2}{S_{u}}_{\infty }+{\left|\delta \frac{\beta }{N}\right|}^{2}I_{\infty }{S_{v}}_{\infty }e^{-\mu \tau }}{|\mu +\rho {|}^{2}{S_{v}}_{\infty }}<1,$ implies


$${\left|f_{2}(S_{u},S_{v},I,R,t)\right|}^{2}<k_{2}\left(1+{\left|S_{v}\right|}^{2}\right).$$

For the function $f_{3}$, we have


$${\left|f_{3}(S_{u},S_{v},I,R,t)\right|}^{2}={\left|\frac{\beta I}{N}S_{u}e^{-\mu \tau }+\delta \frac{\beta I}{N}S_{v}e^{-\mu \tau }+\sigma \lambda R-(\mu +\gamma )I\right|}^{2},$$

$${\left|f_{3}(S_{u},S_{v},I,R,t)\right|}^{2}\leq 2{\left|\frac{\beta I}{N}S_{u}e^{-\mu \tau }\right|}^{2}+2{\left|\delta \frac{\beta I}{N}S_{v}e^{-\mu \tau }\right|}^{2}+2|\sigma \lambda R{|}^{2}+2{\left|(\mu +\gamma )I\right|}^{2},$$

$$\begin{array}{cc} {\left|f_{3}(S_{u},S_{v},I,R,t)\right|}^{2} & \leq 2(\sup_{t\in D_{⋋}}{\left|\frac{\beta I}{N}S_{u}e^{-\mu \tau }\right|}^{2}+\sup_{t\in D_{⋋}}{\left|\delta \frac{\beta I}{N}S_{v}e^{-\mu \tau }\right|}^{2}+\sup_{t\in D_{⋋}}|\sigma \lambda R{|}^{2}\\ & \;+\sup_{t\in D_{⋋}}{\left|(\mu +\gamma )I\right|}^{2}), \end{array}$$

$${\left|f_{3}(S_{u},S_{v},I,R,t)\right|}^{2}\leq 2({\left|\frac{\beta }{N}\right|}^{2}I_{\infty }{S_{u}}_{\infty }e^{-\mu \tau }+{\left|\delta \frac{\beta }{N}\right|}^{2}I_{\infty }{S_{v}}_{\infty }e^{-\mu \tau }+|\sigma \lambda {|}^{2}R_{\infty }+{\left|(\mu +\gamma )I_{\infty }\right|}^{2}),$$

$$\begin{array}{cc} {\left|f_{3}(S_{u},S_{v},I,R,t)\right|}^{2} & \leq 2\left(|\mu +\gamma {|}^{2}I_{\infty }\right)\\ & \;\times \left(1+\frac{{\left|\frac{\beta }{N}\right|}^{2}I_{\infty }{S_{u}}_{\infty }e^{-\mu \tau }+{\left|\delta \frac{\beta }{N}\right|}^{2}I_{\infty }{S_{v}}_{\infty }e^{-\mu \tau }+|\sigma \lambda {|}^{2}R_{\infty }}{\left(|\mu +\gamma {|}^{2}I_{\infty }\right)}\right). \end{array}$$

Under the condition $\frac{{\left|\frac{\beta }{N}\right|}^{2}I_{\infty }{S_{u}}_{\infty }e^{-\mu \tau }+{\left|\delta \frac{\beta }{N}\right|}^{2}I_{\infty }{S_{v}}_{\infty }e^{-\mu \tau }+|\sigma \lambda {|}^{2}R_{\infty }}{\left(|\mu +\gamma {|}^{2}I_{\infty }\right)}<1,$ yields


$${\left|f_{3}(S_{u},S_{v},I,R,t)\right|}^{2}<k_{3}\left(1+|I{|}^{2}\right).$$

For the function $f_{4}$, we have


$${\left|f_{4}(S_{u},S_{v},I,R,t)\right|}^{2}={\left|\gamma I-(\sigma \lambda +\mu )R\right|}^{2},$$

$${\left|f_{4}(S_{u},S_{v},I,R,t)\right|}^{2}\leq |\gamma I{|}^{2}+{\left|(\sigma \lambda +\mu )R\right|}^{2}$$

$${\left|f_{4}(S_{u},S_{v},I,R,t)\right|}^{2}\leq {(sup}_{t\in D_{⋋}}|\gamma I{|}^{2}+\sup_{t\in D_{⋋}}{\left|(\sigma \lambda +\mu )R\right|}^{2}),$$

$${\left|f_{4}(S_{u},S_{v},I,R,t)\right|}^{2}\leq |\gamma {|}^{2}I_{\infty }+{\left|(\sigma \lambda +\mu )\right|}^{2}R_{\infty },$$

$${\left|f_{3}(S_{u},S_{v},I,R,t)\right|}^{2}\leq {\left|(\sigma \lambda +\mu )\right|}^{2}R_{\infty }\left(1+\frac{|\gamma {|}^{2}I_{\infty }}{{\left|(\sigma \lambda +\mu )\right|}^{2}R_{\infty }}\right),$$

The condition $\frac{|\gamma {|}^{2}I_{\infty }}{{\left|(\sigma \lambda +\mu )\right|}^{2}R_{\infty }}<1,$ implies


$${\left|f_{4}(S_{u},S_{v},I,R,t)\right|}^{2}<k_{4}\left(1+|R{|}^{2}\right).$$

Therefore, the condition of linear growth is verified if.


$$\max\left\{\begin{array}{c} \frac{{\left|\frac{\beta }{N}\right|}^{2}{e^{-\mu \tau }I}_{\infty }{S_{u}}_{\infty }+{{|\rho {|}^{2}S}_{v}}_{\infty }}{|\Lambda {|}^{2}+|\mu +\alpha {|}^{2}{S_{u}}_{\infty }},\frac{|\alpha {|}^{2}{S_{u}}_{\infty }+{\left|\delta \frac{\beta }{N}\right|}^{2}I_{\infty }{S_{v}}_{\infty }e^{-\mu \tau }}{|\mu +\rho {|}^{2}{S_{v}}_{\infty }},\\ \frac{{\left|\frac{\beta }{N}\right|}^{2}I_{\infty }{S_{u}}_{\infty }e^{-\mu \tau }+{\left|\delta \frac{\beta }{N}\right|}^{2}I_{\infty }{S_{v}}_{\infty }e^{-\mu \tau }+|\sigma \lambda {|}^{2}R_{\infty }}{\left(|\mu +\gamma {|}^{2}I_{\infty }\right)},\frac{|\gamma {|}^{2}I_{\infty }}{{\left|(\sigma \lambda +\mu )\right|}^{2}R_{\infty }} \end{array}\right\}<1,asdesired.$$

### Analysis of model

This section will briefly discuss the equilibrium of the Scabies delayed model. We will discuss, Scabies free equilibrium $\left(SFE-D_{1}\right)$, and Scabies existing equilibrium $\left(SEE-D_{2}\right)$ given by


$$D_{1}=\left({S_{u}}^{1},{S_{v}}^{1},I^{1},R^{1}\right)=(\frac{\Lambda (\mu +\rho )}{\mu (\mu +\rho +\alpha )},\frac{\alpha \Lambda }{\mu (\mu +\rho +\alpha )},0,0and$$

$$D_{2}=\left({S_{u}}^{*},{S_{v}}^{*},I^{*},R^{*}\right).$$

$${S_{u}}^{*}=A-\delta {S_{v}}^{*},{S_{v}}^{*}=\frac{\alpha A}{\left\{\alpha \delta +\delta \frac{\beta }{N}I^{*}e^{-\mu \tau }+(\mu +\rho )\right\}},I^{*}=\frac{-B_{2}\pm \sqrt{{B_{2}}^{2}-4B_{1}B_{3}}}{2B_{1}},$$

$$R^{*}=\frac{\gamma I^{*}}{\left(\sigma \lambda +\mu \right)}.$$

Where,


$$B_{1}=A\delta {{\left(\frac{\beta }{N}\right)}^{2}e^{-\mu \tau }}^{2},$$

$$B_{2}=A\alpha \delta \frac{\beta }{N}e^{-\mu \tau }-\delta \alpha A\frac{\beta }{N}e^{-\mu \tau }-\Lambda \delta \frac{\beta }{N}e^{-\mu \tau }+A\delta \frac{\beta }{N}e^{-\mu \tau }(\mu +\alpha )+A(\mu +\rho )\frac{\beta }{N}e^{-\mu \tau },$$

$$B_{3}=-\Lambda (\mu +\rho )-\Lambda \alpha \delta -\rho \alpha A+A\alpha \delta (\mu +\alpha )+A(\mu +\rho )(\mu +\alpha )-(\mu +\alpha )\delta \alpha A.$$

For reproduction number, the transmission and transition matrices have been obtained from the given model and denoted by F and G, respectively.


$$F=[\begin{array}{cc} (\frac{\beta }{N}S_{u}e^{-\mu \tau }+\delta \frac{\beta }{N}S_{v}e^{-\mu \tau } & 0\\ 0 & 0 \end{array}],G=[\begin{array}{cc} \mu +\gamma & 0\\ -\gamma & \mu \end{array}].$$

$${FG}^{-1}=[\begin{array}{cc} \left(\frac{\beta }{N}S_{u}e^{-\mu \tau }+\delta \frac{\beta }{N}S_{v}e^{-\mu \tau }\right) & 0\\ 0 & 0 \end{array}][\begin{array}{cc} \frac{1}{\left(\mu +\gamma \right)} & 0\\ \frac{\gamma }{\mu (\mu +\gamma )} & \frac{1}{\mu } \end{array}].$$

Hence, the reproduction number ($R_{0}$) is the largest Eigenvalue of ${FG}^{-1}$, we have:


$$R_{0}=\frac{[\frac{\beta }{N}\varepsilon \left(\mu +\rho +\delta \alpha \right)]e^{-\mu \tau }}{\left(\mu +\gamma \right)},\;\varepsilon =\frac{\Lambda }{\mu (\mu +\rho +\alpha )}.$$

### Stability results

The Jacobian matrix of the system (1–4) and its elements are given below:


$$J_{S_{n}}=[\begin{array}{cccc} J_{11} & J_{12} & J_{13} & J_{14}\\ J_{21} & J_{22} & J_{23} & J_{24}\\ J_{31} & J_{32} & J_{33} & J_{34}\\ J_{41} & J_{42} & J_{43} & J_{44} \end{array}]$$ (5)

$J_{11}=-\frac{\beta }{N}Ie^{-\mu \tau }-(\mu +\alpha )$, $J_{12}=\rho$, $J_{13}=-\frac{\beta }{N}S_{u}e^{-\mu \tau },$ $J_{14}=0$, $J_{21}=\alpha ,J_{22}=-\delta \frac{\beta }{N}Ie^{-\mu \tau }-(\mu +\rho ),J_{23}=-\delta \frac{\beta }{N}S_{v}e^{-\mu \tau }$, $J_{24}=0$, $J_{31}=\frac{\beta }{N}Ie^{-\mu \tau },J_{32}=\delta \frac{\beta }{N}Ie^{-\mu \tau }$, $J_{33}=\frac{\beta }{N}S_{u}e^{-\mu \tau }+\delta \frac{\beta }{N}S_{v}e^{-\mu \tau }-(\mu +\gamma ,J_{34}=\sigma \lambda$, $J_{41}=0$, $J_{42}=0$,$J_{43}=\gamma ,J_{44}=-(\sigma \lambda +\mu ).$

**Theorem 3.** The Scabies-free equilibrium, $D_{1}=\left({S_{u}}^{1},{S_{v}}^{1},I^{1},R^{1}\right)=\left(\frac{\Lambda (\mu +\rho )}{\mu (\mu +\rho +\alpha )},\frac{\alpha \Lambda }{\mu (\mu +\rho +\alpha )},0,0\right)$ is stable asymptotically in the sense of local if $R_{0}<1$.

***Proof*:** The Jacobian matrix (5) at the $D_{1}=\left({S_{u}}^{1},{S_{v}}^{1},I^{1},R^{1}\right)=\left(\frac{\Lambda (\mu +\rho )}{\mu (\mu +\rho +\alpha )},\frac{\alpha \Lambda }{\mu (\mu +\rho +\alpha )},0,0\right)$ is as follows:


$$\mathrm{\backslash setlength}\mathrm{\backslash arraycolsep}\mathrm{-0.5pt}{J_{S_{n}}|}_{D_{1}}=[\begin{array}{cccc} -(\mu +\alpha ) & \rho & -\frac{\frac{\beta }{N}\Lambda (\mu +\rho )}{\mu (\mu +\rho +\alpha )}e^{-\mu \tau } & 0\\ \alpha & -(\mu +\rho ) & -\frac{\delta \frac{\beta }{N}\alpha \Lambda }{\mu (\mu +\rho +\alpha )}e^{-\mu \tau } & 0\\ 0 & 0 & \frac{\frac{\beta }{N}\Lambda (\mu +\rho )}{\mu (\mu +\rho +\alpha )}e^{-\mu \tau }+\frac{\delta \frac{\beta }{N}\alpha \Lambda }{\mu (\mu +\rho +\alpha )}e^{-\mu \tau }-(\mu +\gamma ) & \sigma \lambda \\ 0 & 0 & \gamma & -(\sigma \lambda +\mu ) \end{array}]$$

The detailed proof is given in Appendix A. Therefore, by Routh-Hurwitz Criterion for 4^th^ -degree polynomial, both fixed values of $g_{1}>0,g_{1}g_{2}-{g_{0}g}_{3}>0,\left(g_{1}g_{2}-{g_{0}g}_{3}\right)g_{3}-{g_{1}^{2}g}_{4}>0,g_{4}>0$ if $R_{0}<1.$ Hence, the system’s Scabies-free equilibrium $D_{1}$ is stable asymptotically in the sense of local. In other circumstances, if $R_{0}>1,$ Routh Hurwitz’s condition does not hold. Thus, $D_{1}$ is unstable.

**Theorem 4.** The Scabies existing equilibrium ($S_{n}EE-D_{2}$), $D_{2}=\left({S_{u}}^{*},{S_{v}}^{*},I^{*},R^{*}\right)$ is stable asymptotically in the sense of local if $R_{0}>1$.

***Proof*:** The Jacobian matrix (5) at the $D_{2}=\left({S_{u}}^{*},{S_{v}}^{*},I^{*},R^{*}\right)$ is as follows:


$$\mathrm{\backslash fontsize}68\mathrm{\backslash selectfont}{J_{S_{n}}|}_{D_{2}}=[\begin{array}{cccc} -\frac{\beta }{N}{I^{*}e}^{-\mu \tau }-(\mu +\alpha ) & \rho & -\frac{\beta }{N}{S_{u}}^{*}e^{-\mu \tau } & 0\\ \alpha & -\delta \frac{\beta }{N}I^{*}e^{-\mu \tau }-(\mu +\rho ) & -\delta \frac{\beta }{N}{S_{v}}^{*}e^{-\mu \tau } & 0\\ \frac{\beta }{N}I^{*}e^{-\mu \tau } & \delta \frac{\beta }{N}I^{*}e^{-\mu \tau } & \frac{\beta }{N}{S_{u}}^{*}e^{-\mu \tau }+\delta \frac{\beta }{N}{S_{v}}^{*}e^{-\mu \tau }-(\mu +\gamma ) & \sigma \lambda \\ 0 & 0 & \gamma & –(\sigma \lambda +\mu ) \end{array}]$$

The detailed proof is given in Appendix B. By the Routh-Hurwitz Criterion for 4^th^-degree polynomial; the given constraint has been verified if $R_{0}>1.$ Therefore, the Scabies existing equilibrium ($S_{n}EE-D_{2}$) of the system (1–4) are stable asymptotically in the sense of local.

### Global stability

**Theorem 5.** The Scabies-free equilibrium ($S_{n}$FE-$D_{1}$), $D_{1}=\left({S_{u}}^{1},{S_{v}}^{1},I^{1},R^{1}\right)=(\frac{\Lambda (\mu +\rho )}{\mu (\mu +\rho +\alpha )},\frac{\alpha \Lambda }{\mu (\mu +\rho +\alpha )},0,0)$ stable asymptotically in the sense of global if $R_{0}<1$.

***Proof*:** Define the Volterra Lyapunov function F:B→ℝ defined as


$$F(I)=\ln\left(\frac{I}{I^{1}}\right).$$

$$\frac{dF(I)}{dt}=\frac{1}{I}\frac{dI}{dt}.$$

$$\frac{dF(I)}{dt}=\frac{1}{I}\left(\frac{\beta }{N}IS_{u}e^{-\mu \tau }+\delta \frac{\beta }{N}IS_{v}e^{-\mu \tau }+\sigma \lambda R-(\mu +\gamma )I\right).$$

$$\frac{dF(I)}{dt}=\left(\frac{\beta }{N}S_{u}e^{-\mu \tau }+\delta \frac{\beta }{N}S_{v}e^{-\mu \tau }+\sigma \lambda R-(\mu +\gamma )\right).$$

After putting the value of $D_{1}$, we have,


$$\frac{dF(I)}{dt}\leq \left(\frac{\beta }{N}{S_{u}}^{1}e^{-\mu \tau }+\delta \frac{\beta }{N}{S_{v}}^{1}e^{-\mu \tau }-(\mu +\gamma )\right).$$

$$\frac{dF(I)}{dt}\leq (\mu +\gamma )\left(\frac{\frac{\beta }{N}{S_{u}}^{1}e^{-\mu \tau }+\delta \frac{\beta }{N}{S_{v}}^{1}e^{-\mu \tau }}{\left(\mu +\gamma \right)}-1\right)=(\mu +\gamma )\left(R_{0}-1\right).$$

Since, $\frac{dF(I)}{dt}\leq 0$, for $R_{0}<1$. Therefore, $D_{1}$ is stable asymptotically in the global sense.

**Theorem 6.** The Scabies existence equilibrium (SEE-$D_{2}$), $D_{2}=\left({S_{u}}^{*},{S_{v,}}^{*},I^{*},R^{*}\right)$ is stable asymptotically in the sense of global if $R_{0}>1$.

***Proof:*** Define the Volterra Lyapunov function G:B→ℝ define as


$$G=(S_{u}-S_{u}^{*}-S_{u}^{*}\log\frac{S_{u}}{S_{u}^{*}}!+\;\left(S_{v}-S_{v}^{*}-S_{u}^{*}\log\frac{S_{v}}{S_{v}^{*}}\right)\;+\;\left(I-I^{*}-I^{*}\log\frac{I}{I^{*}}\right)+\left(R-R^{*}-R^{*}\log\frac{R}{R^{*}}\right).$$

$$\frac{dG}{dt}=\left(1-\frac{S_{u}^{*}}{S_{u}}\right)\frac{dS_{u}}{dt}+\left(1-\frac{S_{V}^{*}}{S_{V}}\right)\frac{dS_{v}}{dt}+\left(1-\frac{I^{*}}{I}\right)\frac{dI}{dt}+\left(1-\frac{R^{*}}{R}\right)\frac{dR}{dt}.$$

$$\frac{dG}{dt}=-\left(\frac{{\left(S_{u}{-S}_{u}^{*}\right)}^{2}\Lambda }{S_{u}S_{u}^{*}}\right)-\left(\frac{{\left(S_{V}{-S}_{V}^{*}\right)}^{2}\alpha S_{u}}{S_{V}S_{V}^{*}}\right)-\left(\frac{{\left({I-I}^{*}\right)}^{2}\sigma \lambda R}{{II}^{*}}\right)-\left(\frac{{\left(R-R^{*}\right)}^{2}\gamma I}{RR^{*}}\right).$$

Since, $\frac{dG}{dt}\leq 0$, for $R_{0}>1$ and $\frac{dG}{dt}=0$ only if $S_{u}=S_{u}^{*}$, $S_{v}=S_{v}^{*}$, $I=I^{*}$ and $R=R^{*}$. Hence, by Lasalle’s invariance principle, $D_{2}$ is stable asymptotically in the global sense.

### Second-order Lyapunov stability

**Theorem 7.** The system (1–4) at $D_{1}=\left({S_{u,}}^{1},{S_{v}}^{1},I^{1},R^{1}\right)=(\frac{\Lambda (\mu +\rho )}{\mu (\mu +\rho +\alpha )},\frac{\alpha \Lambda }{\mu (\mu +\rho +\alpha )},0,0)$ is globally asymptotical stable (GAS) if $R_{0}<1$.

**Proof:** Consider,


$$U^{\prime }(I)=\frac{1}{I}\frac{dI}{dt}.$$

$$U\prime \left(I\right)=\frac{1}{I}\frac{dI}{dt}.$$

$$U\prime \prime \left(I\right)=\frac{1}{I}\frac{d}{dt}\left(\frac{dI}{dt}\right)-\frac{1}{I^{2}}{\left(\frac{dI}{dt}\right)}^{2}.$$

$$\begin{array}{cc} U^{\prime \prime }(I) & \leq \frac{1}{I}\frac{d}{dI}\left(\frac{\beta }{N}IS_{u}e^{-\mu \tau }+\delta \frac{\beta }{N}IS_{v}e^{-\mu \tau }-(\mu +\gamma )I\right)\frac{dI}{dt}\\ & \;-{\left(\frac{\beta }{N}S_{u}e^{-\mu \tau }+\delta \frac{\beta }{N}S_{v}e^{-\mu \tau }+\frac{\sigma \lambda R}{I}-(\mu +\gamma )\right)}^{2}. \end{array}$$

$$U^{\prime \prime }(I)\leq {\left((\mu +\gamma )\right)}^{2}{\left(1-\frac{\frac{\beta }{N}\Lambda (\mu +\rho )e^{-\mu \tau }+\delta \frac{\beta }{N}\alpha \Lambda e^{-\mu \tau }}{\mu (\mu +\rho +\alpha )(\mu +\gamma )}\right)}^{2}.$$

$$U^{\prime \prime }(I)\leq -{\left((\mu +\gamma )\right)}^{2}{\left(1-R_{0}\right)}^{2}.$$

$$U^{\prime \prime }(I)\leq 0,forR_{0}<1.$$

Hence, the system is globally asymptotically stable at $D_{1}$.

**Theorem 8.** (Global stability at $D_{2}$) The system (1–4) at $D_{2}=\left({S_{u}}^{*},{S_{v,}}^{*},I^{*},R^{*}\right)$ is globally asymptotically stable (GAS) if $R_{0}>1$.

**Proof:** Consider


$$Z^{\prime \prime }=\frac{d}{dt}\left(\frac{S_{u}-{S_{u}}^{*}}{S_{u}}\right)\frac{dS_{u}}{dt}+\frac{d}{dt}\left(\frac{S_{v}-{S_{v}}^{*}}{S_{v}}\right)\frac{dS_{v}}{dt}+\frac{d}{dt}\left(\frac{I-I^{*}}{I}\right)\frac{dI}{dt}+\frac{d}{dt}\left(\frac{R-R^{*}}{R}\right)\frac{dR}{dt}.$$

$$\begin{array}{cc} Z^{\prime \prime } & =\frac{{S_{u}}^{*}}{{S_{u}}^{2}}{\left(\frac{dS_{u}}{dt}\right)}^{2}+\left(\frac{S_{u}-{S_{u}}^{*}}{S_{u}}\right)\frac{d^{2}S}{dt^{2}}+\frac{{S_{v}}^{*}}{{S_{v}}^{2}}{\left(\frac{dS_{v}}{dt}\right)}^{2}+\left(\frac{S_{v}-{S_{v}}^{*}}{S_{v}}\right)\frac{d^{2}S_{v}}{dt^{2}}+\frac{I^{*}}{I^{2}}{\left(\frac{dI}{dt}\right)}^{2}\\ & \;+\left(\frac{I-I^{*}}{I}\right)\frac{d^{2}I}{dt^{2}}+\frac{R^{*}}{R^{2}}{\left(\frac{dR}{dt}\right)}^{2}+\left(\frac{R-R^{*}}{R}\right)\frac{d^{2}R}{dt^{2}}. \end{array}$$

$$\begin{array}{cc} Z^{\prime \prime } & =\frac{{S_{u}}^{*}}{{S_{u}}^{2}}{\left(\Lambda +\rho S_{v}-\frac{\beta }{N}IS_{u}e^{-\mu \tau }-(\mu +\alpha )S_{u}\right)}^{2}+\frac{{S_{v}}^{*}}{{S_{v}}^{2}}{\left(\alpha S_{u}-\delta \frac{\beta }{N}IS_{v}e^{-\mu \tau }-(\mu +\rho )S_{v}\right)}^{2}\\ & \;+\frac{I^{*}}{I^{2}}{\left(\frac{\beta }{N}IS_{u}e^{-\mu \tau }+\delta \frac{\beta }{N}IS_{v}e^{-\mu \tau }+\sigma \lambda R-(\mu +\gamma )I\right)}^{2}+\frac{R^{*}}{R^{2}}{\left(\gamma I-(\sigma \lambda +\mu )R\right)}^{2}\\ & \;+\left(1-\frac{{S_{u}}^{*}}{S_{u}}\right)\left(-\frac{\beta }{N}Ie^{-\mu \tau }-(\mu +\alpha )\right)\left(\Lambda +\rho S_{v}-\frac{\beta }{N}IS_{u}e^{-\mu \tau }-(\mu +\alpha )S_{u}\right)\\ & \;+\frac{d}{dS_{v}}\left(-\delta \frac{\beta }{N}Ie^{-\mu \tau }-(\mu +\rho )\right)\left(\alpha S_{u}-\delta \frac{\beta }{N}IS_{v}e^{-\mu \tau }-(\mu +\rho )S_{v}\right)\\ & \;+\left(1-\frac{I^{*}}{I}\right)\frac{d}{dI}\left(\frac{\beta }{N}S_{u}e^{-\mu \tau }+\delta \frac{\beta }{N}S_{v}e^{-\mu \tau }-(\mu +\gamma )\right)\\ & \;\times \left(\frac{\beta }{N}IS_{u}e^{-\mu \tau }+\delta \frac{\beta }{N}IS_{v}e^{-\mu \tau }+\sigma \lambda R-(\mu +\gamma )I\right)\\ & \;+\left(1-\frac{R^{*}}{R}\right)\frac{d}{dR}\left(-(\sigma \lambda +\mu )\right)\left(\gamma I-(\sigma \lambda +\mu )R\right). \end{array}$$

For Simplicity, we choose $Z^{\prime \prime }=\phi_{1}-\phi_{2}$.


$$\begin{array}{cc} \phi_{1} & =\frac{{S_{u}}^{*}}{{S_{u}}^{2}}{(\Lambda }^{2}+\Lambda \rho S_{v}-\Lambda \frac{\beta }{N}IS_{u}e^{-\mu \tau }-\Lambda (\mu +\alpha )S_{u}\\ & \;+\Lambda \rho S_{v}+({\rho S_{v})}^{2}-\rho \frac{\beta }{N}IS_{u}{S_{v}e}^{-\mu \tau }-\rho (\mu +\alpha )S_{u}S_{v}\\ & \;-\Lambda \frac{\beta }{N}IS_{u}e^{-\mu \tau }-\frac{\beta }{N}I\rho S_{v}S_{u}e^{-\mu \tau }{+\left(\frac{\beta }{N}IS_{u}e^{-\mu \tau }\right)}^{2}\\ & \;+(\mu +\alpha )\frac{\beta }{N}I({S_{u})}^{2}e^{-\mu \tau }-\Lambda (\mu +\alpha )S_{u}-\rho (\mu +\alpha )S_{u}S_{v}\\ & \;+(\mu +\alpha )\frac{\beta }{N}I{{(S}_{u})}^{2}e^{-\mu \tau }+{\left(\mu +\alpha ){(S}_{u}\right)}^{2})\\ & \;+\frac{{S_{v}}^{*}}{{S_{v}}^{2}}({\left(\alpha S_{u}\right)}^{2}-\alpha \delta \frac{\beta }{N}IS_{u}S_{v}e^{-\mu \tau }-\alpha S_{u}(\mu +\rho ){S_{u}S}_{v}\\ & \;-\alpha \delta \frac{\beta }{N}I{S_{u}S}_{v}e^{-\mu \tau }+{\left(\delta \frac{\beta }{N}IS_{v}e^{-\mu \tau }\right)}^{2}\\ & \;+\delta \frac{\beta }{N}I(\mu +\rho )S_{v}S_{v}e^{-\mu \tau }-(\mu +\rho )\alpha S_{u}S_{v}\\ & \;+(\mu +\rho )\delta \frac{\beta }{N}I\left({S_{v})}^{2}e^{-\mu \tau }+{\left(\mu +\rho ){(S}_{v}\right)}^{2}\right)\\ & \;+\frac{I^{*}}{I^{2}}({\frac{\beta }{N}IS_{u}e^{-\mu \tau })}^{2}+S_{u}\delta {\left(\frac{\beta }{N}I\right)}^{2}S_{u}S_{v}e^{-2\mu \tau }\\ & \;+\sigma \lambda \frac{\beta }{N}IS_{u}Re^{-\mu \tau }-(\mu +\gamma )\frac{\beta }{N}I^{2}S_{u}e^{-\mu \tau }\\ & \;+\delta {\left(\frac{\beta }{N}I\right)}^{2}S_{u}S_{v}e^{-2\mu \tau }+{\left(\delta \frac{\beta }{N}IS_{v}e^{-\mu \tau }\right)}^{2}\\ & \;+\sigma \lambda \delta \frac{\beta }{N}IS_{v}Re^{-\mu \tau }-(\mu +\gamma )\delta \frac{\beta }{N}I^{2}S_{v}e^{-\mu \tau }\\ & \;+\sigma \lambda \frac{\beta }{N}I{RS}_{u}e^{-\mu \tau }+\sigma \lambda \delta \frac{\beta }{N}IRS_{v}e^{-\mu \tau }+(\sigma \lambda R{)}^{2}\\ & \;-\sigma \lambda (\mu +\gamma )IR-(\mu +\gamma )\frac{\beta }{N}I^{2}S_{u}e^{-\mu \tau }-(\mu +\gamma )\delta \frac{\beta }{N}I^{2}S_{v}e^{-\mu \tau }\\ & \;-\sigma \lambda (\mu +\gamma )IR+{\left((\mu +\gamma )I\right)}^{2})+\frac{R^{*}}{R^{2}}{\left((\gamma I\right)}^{2}\\ & \;-(\sigma \lambda +\mu )\gamma IR-(\sigma \lambda +\mu )\gamma IR+{\left((\sigma \lambda +\mu )R\right)}^{2}). \end{array}$$

$$\begin{array}{cc} \phi_{2} & =\{\left(-\Lambda \frac{\beta }{N}Ie^{-\mu \tau }-\frac{\beta }{N}\rho S_{v}Ie^{-\mu \tau }+{\left(\frac{\beta }{N}I\right)}^{2}S_{u}e^{-2\mu \tau }+\frac{\beta }{N}(\mu +\alpha )S_{u}Ie^{2\mu \tau }\right)\\ & \;\times (-\Lambda (\mu +\alpha )-(\mu +\alpha )\rho S_{v}+(\mu +\alpha )\frac{\beta }{N}IS_{u}e^{-\mu \tau }+(\mu +\alpha {)}^{2}S_{u}\\ & \;+-\alpha \delta \frac{\beta }{N}IS_{u}e^{-\mu \tau }+{\left(\delta \frac{\beta }{N}I\right)}^{2}S_{v}e^{-2\mu \tau }+\delta \frac{\beta }{N}(\mu +\rho )S_{v}Ie^{-\mu \tau }\\ & \;-(\mu +\rho )\alpha S_{u}+(\mu +\rho )\delta \frac{\beta }{N}IS_{v}e^{-\mu \tau }+(\mu +\rho {)}^{2}S_{v}+{\left(\frac{\beta }{N}S_{u}\right)}^{2}Ie^{-2\mu \tau }\\ & \;+\delta {\left(\frac{\beta }{N}\right)}^{2}IS_{u}S_{v}e^{-2\mu \tau }+\sigma \frac{\beta }{N}S_{u}Re^{-\mu \tau }-(\mu +\gamma )I\frac{\beta }{N}S_{u}e^{-\mu \tau }\\ & \;+\delta {\left(\frac{\beta }{N}\right)}^{2}IS_{v}S_{u}e^{-2\mu \tau }+{\left(\delta \beta \frac{\beta }{N}\right)}^{2}Ie^{-2\mu \tau }+\delta \frac{\beta }{N}\sigma \lambda RS_{v}e^{-\mu \tau }\\ & \;-\delta \frac{\beta }{N}{(\mu +\gamma )IS}_{v}e^{-\mu \tau }-(\mu +\gamma )\frac{\beta }{N}IS_{u}e^{-\mu \tau }-(\mu +\gamma )\delta \frac{\beta }{N}IS_{v}e^{-\mu \tau }\\ & \;-(\mu +\gamma )\sigma \lambda R+(\mu +\gamma {)}^{2}I-(\sigma \lambda +\mu )\gamma I+(\sigma \lambda +\mu {)}^{2}R)\\ & \;+\left(-\Lambda \frac{\beta }{N}Ie^{-\mu \tau }-\frac{\beta }{N}\rho S_{v}Ie^{-\mu \tau }+{\left(\frac{\beta }{N}I\right)}^{2}S_{u}e^{-2\mu \tau }+\frac{\beta }{N}(\mu +\alpha )S_{u}Ie^{-\mu \tau }\right)\\ & \;\times (-\Lambda (\mu +\alpha )-(\mu +\alpha )\rho S_{v}+(\mu +\alpha )\frac{\beta }{N}IS_{u}e^{-\mu \tau }+(\mu +\alpha {)}^{2}S_{u}\\ & \;-\alpha \delta \frac{\beta }{N}IS_{u}e^{-\mu \tau }+{\left(\delta \frac{\beta }{N}I\right)}^{2}S_{v}e^{-2\mu \tau }+\delta \frac{\beta }{N}(\mu +\rho )S_{v}Ie^{-\mu \tau }\\ & \;-(\mu +\rho )\alpha S_{u}+(\mu +\rho )\delta \frac{\beta }{N}IS_{v}e^{-\mu \tau }+(\mu +\rho {)}^{2}S_{v}+{\left(\frac{\beta }{N}S_{u}\right)}^{2}Ie^{-2\mu \tau }\\ & \;+\delta {\left(\frac{\beta }{N}\right)}^{2}IS_{u}S_{v}e^{-2\mu \tau }+\sigma \lambda \frac{\beta }{N}S_{u}Re^{-\mu \tau }-(\mu +\gamma )I\frac{\beta }{N}S_{u}e^{-\mu \tau }+\delta {\left(\frac{\beta }{N}\right)}^{2}IS_{v}S_{u}e^{-2\mu \tau }\\ & \;+{\left(\delta \frac{\beta }{N}S_{v}\right)}^{2}Ie^{-2\mu \tau }+\delta \frac{\beta }{N}\sigma \lambda RS_{v}e^{-\mu \tau }-\delta \frac{\beta }{N}{(\mu +\gamma )IS}_{v}e^{-\mu \tau }\\ & \;-(\mu +\gamma )\frac{\beta }{N}IS_{u}e^{-\mu \tau }-(\mu +\gamma )\delta \frac{\beta }{N}IS_{v}e^{-\mu \tau }-(\mu +\gamma )\sigma \lambda R\\ & \;+(\mu +\gamma {)}^{2}I-(\sigma \lambda +\mu )\gamma I+(\sigma \lambda +\mu {)}^{2}R)\\ & \;+\frac{{S_{u}}^{*}}{S_{u}}\left(-\Lambda \frac{\beta }{N}Ie^{-\mu \tau }-\frac{\beta }{N}\rho S_{v}Ie^{-\mu \tau }+{\left(\frac{\beta }{N}I\right)}^{2}S_{u}e^{-2\mu \tau }+\frac{\beta }{N}(\mu +\alpha )S_{u}Ie^{-\mu \tau }\right)\\ & \;\times (-\Lambda (\mu +\alpha )-(\mu +\alpha )\rho S_{v}+(\mu +\alpha )\frac{\beta }{N}IS_{u}e^{-\mu \tau }+(\mu +\alpha {)}^{2}S_{u})\\ & \;+\frac{{S_{v}}^{*}}{S_{v}}(-\alpha \delta \frac{\beta }{N}IS_{u}e^{-\mu \tau }+{\left(\delta \frac{\beta }{N}I\right)}^{2}S_{v}e^{-2\mu \tau }+\delta \frac{\beta }{N}(\mu +\rho )S_{v}Ie^{-\mu \tau }\\ & \;-(\mu +\rho )\alpha S_{u}+(\mu +\rho )\delta \frac{\beta }{N}IS_{v}e^{-\mu \tau }+(\mu +\rho {)}^{2}S_{v})\\ & \;+\frac{I^{*}}{I}({\left(\frac{\beta }{N}S_{u}\right)}^{2}Ie^{-2\mu \tau }+\delta {\left(\frac{\beta }{N}\right)}^{2}IS_{u}S_{v}e^{-2\mu \tau }+\sigma \lambda \frac{\beta }{N}S_{u}Re^{-\mu \tau }\\ & \;-(\mu +\gamma )I\frac{\beta }{N}S_{u}e^{-\mu \tau }+\delta {\left(\frac{\beta }{N}\right)}^{2}IS_{v}S_{u}e^{-2\mu \tau }+{\left(\delta \frac{\beta }{N}S_{v}\right)}^{2}Ie^{-2\mu \tau }\\ & \;+\delta \frac{\beta }{N}\sigma RS_{v}e^{-\mu \tau }-\delta \frac{\beta }{N}{(\mu +\gamma )IS}_{v}e^{-\mu \tau }-(\mu +\gamma )\frac{\beta }{N}IS_{u}e^{-\mu \tau }\\ & \;-(\mu +\gamma )\delta \frac{\beta }{N}IS_{v}e^{-\mu \tau })+\frac{R^{*}}{R}(-(\mu +\gamma )\sigma \lambda R+(\mu +\gamma {)}^{2}I\\ & \;-(\sigma \lambda +\mu )\gamma I+(\sigma \lambda +\mu {)}^{2}R) \end{array}$$

It can be seen that

$\phi_{1}>\phi_{2}$, $Z^{\prime \prime }>0$.

$\phi_{1}<\phi_{2}$, $Z^{\prime \prime }<0$.

$\phi_{1}=\phi_{2}$, $Z^{\prime \prime }=0$.

### Parameters sensitivity

The sensitivity analysis of the scabies delayed epidemic model shows how changes in key factors, like how fast it spreads how long it takes to show up, and how well vaccination work, can have a big impact on how the disease moves and how we control it. Even small tweaks to these factors can change the basic reproduction number, which might push the system from getting rid of the disease to letting it stick around. To get a handle on this sensitivity is essential to come up with targeted plans and make public health strategies better, so we can keep scabies outbreaks in check. In this section, we employ derivative-based local methods for sensitivity analysis, extracting partial derivatives of outputs concerning inputs, as demonstrated. The study underscores the significance of transmission rates in altering dynamics, transitioning from being Scabies-free to the presence of Scabies.


$$S_{n_{\Lambda }}=\frac{\frac{\partial {ℛ}_{0}}{{ℛ}_{0}}}{\frac{\partial \Lambda }{\Lambda }}=\frac{\Lambda }{{ℛ}_{0}}\times \frac{\partial {ℛ}_{0}}{\partial \Lambda }=1>0,$$

$$S_{n_{\beta }}=\frac{\frac{\partial {ℛ}_{0}}{{ℛ}_{0}}}{\frac{\partial \beta }{\beta }}=\frac{\beta }{{ℛ}_{0}}\times \frac{\partial {ℛ}_{0}}{\partial \beta }=1>0,$$

$$S_{n_{\rho }}=\frac{\frac{\partial {ℛ}_{0}}{{ℛ}_{0}}}{\frac{\partial \rho }{\rho }}=\frac{\rho }{{ℛ}_{0}}\times \frac{\partial {ℛ}_{0}}{\partial \rho }=\frac{\left[\Lambda \frac{\beta }{N}(\mu +\gamma )\right]e^{-\mu \tau }[\alpha -\delta \alpha ]}{[(\mu +\rho +\alpha )(\mu +\gamma ){]}^{2}}\times \frac{\rho }{\frac{[\Lambda \frac{\beta }{N}(\mu +\rho +\delta \alpha )]e^{-\mu \tau }}{\left(\mu +\rho +\alpha )(\mu +\gamma \right)}}>0,$$

$$S_{n_{\alpha }}=\frac{\frac{\partial {ℛ}_{0}}{{ℛ}_{0}}}{\frac{\partial \alpha }{\alpha }}=\frac{\alpha }{{ℛ}_{0}}\times \frac{\partial {ℛ}_{0}}{\partial \alpha }=\frac{\left[\Lambda \frac{\beta }{N}\delta (\mu +\gamma )\right]e^{-\mu \tau }[\alpha -\delta \alpha ]}{[(\mu +\rho +\alpha )(\mu +\gamma ){]}^{2}}\times \frac{\alpha }{\frac{[\Lambda \frac{\beta }{N}(\mu +\rho +\delta \alpha )]e^{-\mu \tau }}{\left(\mu +\rho +\alpha )(\mu +\gamma \right)}}>0,$$

$$S_{n_{\delta }}=\frac{\frac{\partial {ℛ}_{0}}{{ℛ}_{0}}}{\frac{\partial \delta }{\delta }}=\frac{\delta }{{ℛ}_{0}}\times \frac{\partial {ℛ}_{0}}{\partial \delta }=\frac{\Lambda \frac{\beta }{N}\alpha e^{-\mu \tau }}{\left(\mu +\rho +\alpha )(\mu +\gamma \right)}\times \frac{\delta }{\frac{[\Lambda \frac{\beta }{N}(\mu +\rho +\delta \alpha )]e^{-\mu \tau }}{\left(\mu +\rho +\alpha )(\mu +\gamma \right)}}>0,$$

$$S_{n_{\gamma }}=\frac{\frac{\partial {ℛ}_{0}}{{ℛ}_{0}}}{\frac{\partial \gamma }{\gamma }}=\frac{\gamma }{{ℛ}_{0}}\times \frac{\partial {ℛ}_{0}}{\partial \gamma }=\frac{\Lambda \frac{\beta }{N}(\mu +\rho +\delta \alpha )(\mu +\rho +\alpha )e^{-\mu \tau }}{[(\mu +\rho +\alpha )(\mu +\gamma ){]}^{2}}\times \frac{\gamma }{\frac{[\Lambda \frac{\beta }{N}(\mu +\rho +\delta \alpha )]e^{-\mu \tau }}{\left(\mu +\rho +\alpha )(\mu +\gamma \right)}}>0,$$

$$\begin{array}{cc} S_{n_{\mu }} & =\frac{\frac{\partial {ℛ}_{0}}{{ℛ}_{0}}}{\frac{\partial \mu }{\mu }}=\frac{\mu }{{ℛ}_{0}}\times \frac{\partial {ℛ}_{0}}{\partial \mu }\\ & =\frac{-\Lambda \frac{\beta }{N}e^{-\mu \tau }[\tau (\mu +\rho +\alpha )(\mu +\gamma )+(\mu +\rho +\delta \alpha )(2\mu +\gamma +\rho +\alpha )]}{[(\mu +\rho +\alpha )(\mu +\gamma ){]}^{2}}\\ & \;\times \frac{\mu }{\frac{[\Lambda \frac{\beta }{N}(\mu +\rho +\delta \alpha )]e^{-\mu \tau }}{\left(\mu +\rho +\alpha )(\mu +\gamma \right)}}\\ & <0. \end{array}$$

**Fig 2:**
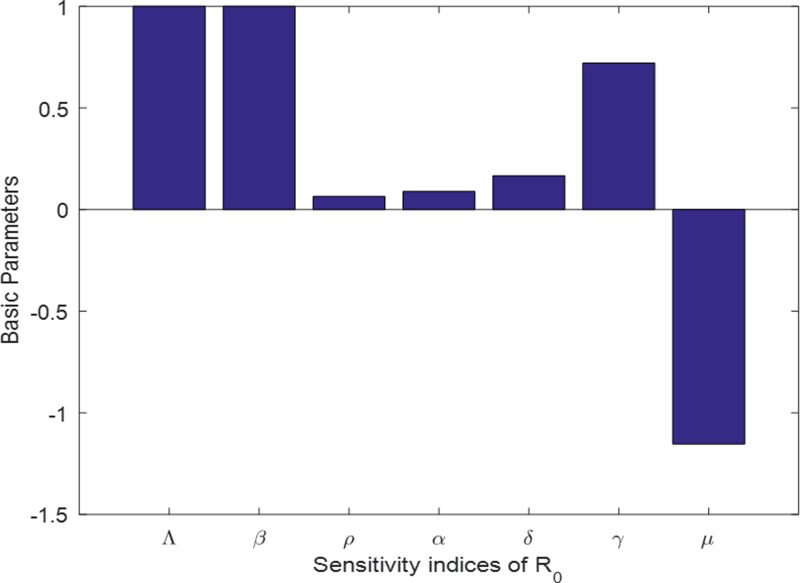
Sensitivity index of parameters.

Subsequently, based on the results above, the conclusion drawn is that Λ,β,ρ,α,δ, and γ exhibit more sensitivity, which means that a small increase in the value of parameters may switch from Scabies free equilibrium to Scabies existing equilibrium and vice versa. While μ is less sensitive, it means that the change in its value does not affect the dynamics of Scabies disease (see Fig 2).

## Results and discussion

**Fig 3:**
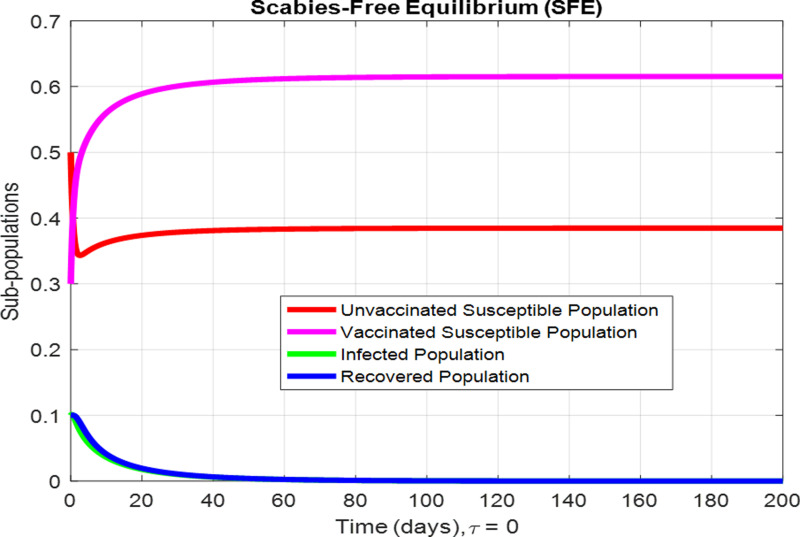
Subpopulations denote the sizes of various groups within the population over time. In this instance, the subpopulations pertain to the dimensions of the scabies-free equilibrium within the model when τ = 0.

**Fig 4:**
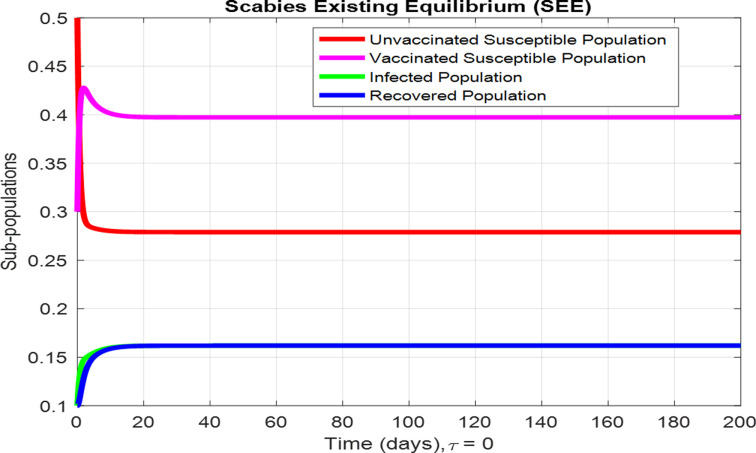
Time plots of each subpopulation at Scabies existing equilibrium when τ=0.

**Fig 5:**
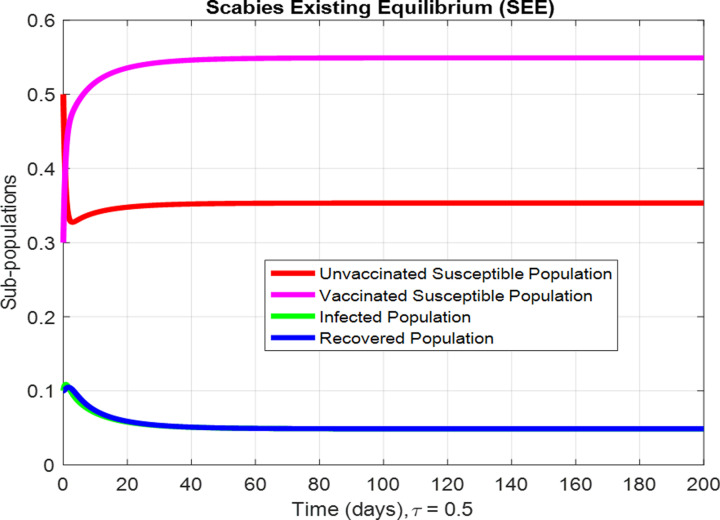
The behaviour of subpopulations at Scabies existing equilibrium when the value of delay parameter is τ=0.5.

**Fig 6:**
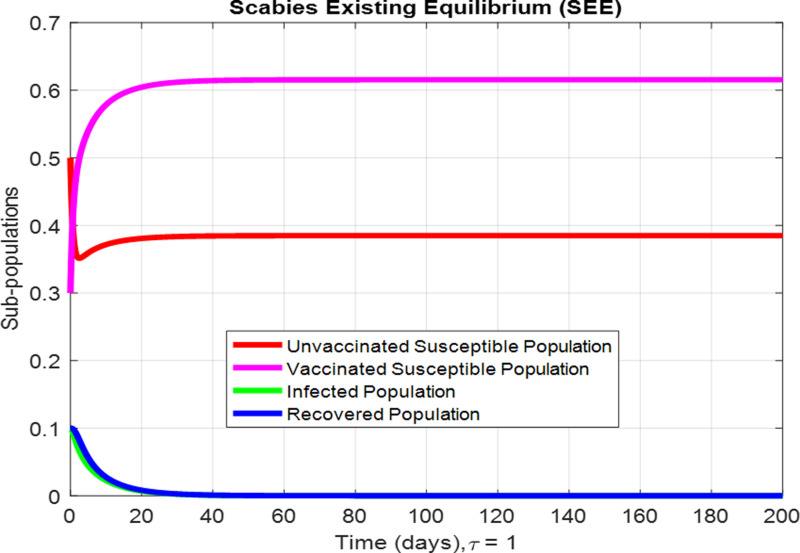
The behaviour of subpopulations at Scabies existing equilibrium when the value of delay parameter is τ=1..

**Fig 7:**
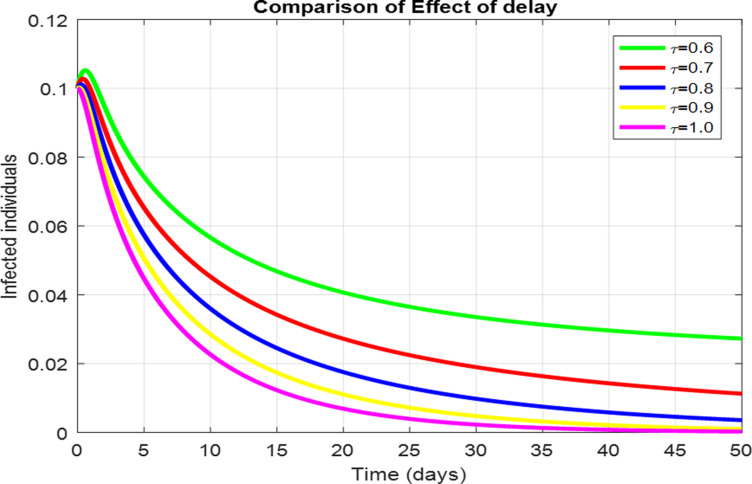
The effect of delay parameters on the infected individuals.

**Fig 8:**
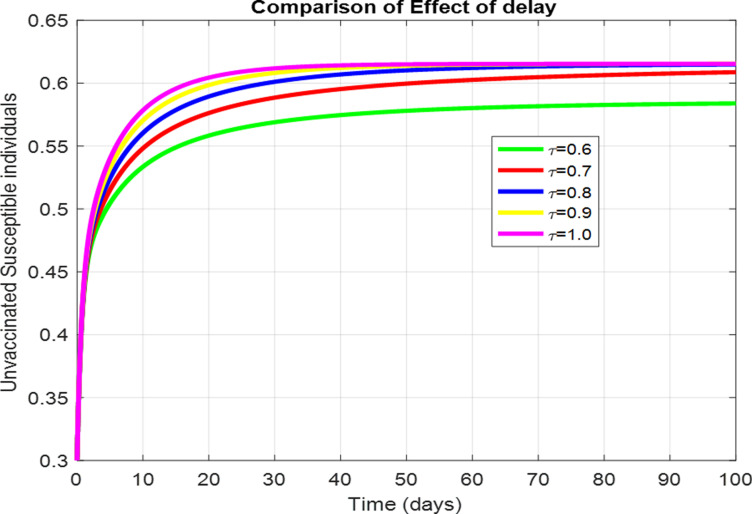
The effect of delay parameters on the unvaccinated susceptible individuals.

**Fig 9:**
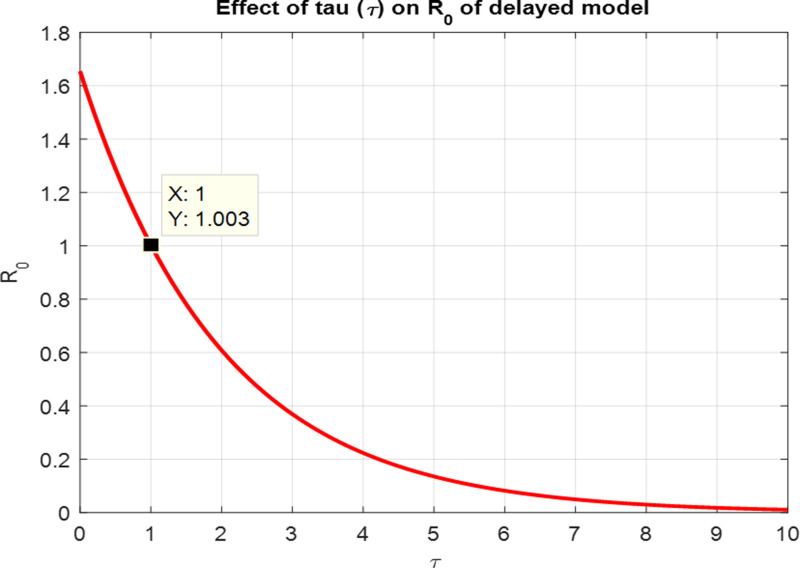
Comparison of the reproduction number with a delay parameter.

For the numerical results of the scabies delayed model (1–4), we have used the Euler method for the given delay differential equations (DDEs) and the parameter values that are taken from scientific literature and presented in Table 1. Fig 3 exhibits the solution, at the Scabies-free equilibrium ($S_{n}$FE-$D_{1})$, when τ=0, that is $D_{1}=\left(S_{u}^{1},S_{v}^{1},I^{1},R^{1}\right)=(0.6163,0.3847,0,0$and the value of the reproduction number is less than one. Furthermore in Fig 3, we have plotted a graphical representation of the system (1–4), by assuming the value of delay is zero to observe the steady state of the system like scabies-free equilibrium and its immense pleasure that the graphical behavior converges to the same equilibria that we have obtained analytically. This act is guaranteed by the true analysis of the given system. Fig 4 displays the solution of the system at Scabies existing equilibrium ($S_{n}$EE-$D_{2})$ when τ=0, that is $D_{2}$ and the value of the reproduction number is greater than one. Figs 5 and 6 show the impact of the effective use of delay tactics like vaccinations in the system. It has been observed that the strain of scabies disease reduces and switches from scabies existing equilibrium to scabies-free equilibrium gradually. Moreover, precautionary may control a disease in a population instead of controlling any other parameters of the model. Furthermore, in Fig 4, we have observed the graphical representation of the system (1–4), by assuming the value of delay is zero. So, we observed that each subpopulation of the system converges to the scabies existing equilibrium of the systems as we have obtained from the model. After that, due to the delayed modeling of the system, we observed the effect of delay on each subpopulation of the model. In Fig 5, we consider the effect of delay instead of perturbed any other parameters of the model, in conclusion, the infectivity of scabies is decreased as we compared with Fig 4. In the same way, we have increased more value of delay tactics in the model and we observed in Fig 6, that each subpopulation of the model shows the same behavior as Fig 3 like scabies-free equilibrium. It means that the effective use of delay tactics in the model is that the dynamics switch from endemic equilibrium to disease-free behavior of disease. Fig 7 (behaviour of infective class at the different delay parameter values) shows the behaviour of an infective class at the different delay parameter values. We observed that at the increased value of delay, the infectivity has been reduced and even converges to zero. Furthermore, the value decreases change the dynamics of the system of Scabies disease from depicting effects of delay on infective individuals—the different values of τ exhibit that the infective class of people reduces even death in the population. Fig 8 (Behaviour of unvaccinated class at the different delay parameter values) represented that with the increase in the delay parameter the susceptibility of humans increases and the infectivity of humans decreases and vice versa. Fig 9 shows the dynamics of the reproduction number concerning the effectiveness of delay tactics. Moreover, for which value of delay, the disease will be endemic in the population or disease-free in a population. Furthermore, the incorporation of delay in this model captures critical time-dependent phenomena such as incubation periods, delayed immune responses, or the effect of interventions. These factors significantly influence the dynamics of the scabies spread, stabilizing the oscillatory behavior in population interactions or prolonging the infection peak.

**Table 1:** Values of parameters.

Parameters	Values (range)/Source [1]
Λ	0.029 ${yr}^{-1}\times 10000$
α	0.8${yr}^{-1}$
ρ	0.0002${yr}^{-1}$
δ	0.125 Assumed
β	0.043${yr}^{-1}$
γ	0.75${yr}^{-1}$
σ	0.25 Assumed
μ	0.02${yr}^{-1}$
τ	≥0

## Conclusion

In this article, we studied a mathematical model of Scabies with a delay effect and stability of the model around equilibria for the second order. The model is based on four sub-populations: susceptible individuals with no vaccination, susceptible individuals with vaccination, infected individuals, and recovered individuals. The contributing task is to analyze the impact of parameters on the dynamics of diseases and their stability. The study proves a positive and boundedness of the model and has two equilibria: a scabies-free equilibrium and a scabies-existing equilibrium. The scabies-free equilibrium is stable if the reproduction number is less than one. In the same line, the scabies-existing equilibrium is stable if the value of the reproduction number is greater than one. We also found that the delay parameters most significantly impacted the model dynamics. Finally, we used the Lyapunov theory and the Lassalle invariance principle to show that the scabies-free equilibrium is globally stable. The article studied a mathematical model of Scabies to determine how the disease spreads and how it can be controlled. The model found that delay tactics are the most effective way to control Scabies. Also, the second-order stability gave us a better grasp of the system’s nonlinearities, which led to a more accurate estimate of how diseases spread in real life. This research adds to the ongoing work in mathematical epidemiology to create reliable models. These models can help shape public health strategies when it comes to planning ways to control scabies in groups that are at high risk. The spatial heterogeneity, stochastic factors, and intervention measures may be considered in future research. This study is a further step towards modeling infectious disease transmission dynamics, and informing public health interventions.

## Appendix A


$${J_{S_{n}}|}_{D_{1}}=[\begin{array}{cccc} -A_{0} & \rho & -A_{2} & 0\\ \alpha & -A_{1} & -A_{3} & 0\\ 0 & 0 & +A_{2}+A_{3}-A_{4} & \sigma \\ 0 & 0 & \gamma & -A_{5} \end{array}]$$

Where, $A_{0}=(\mu +\alpha )$, $A_{1}=$ (μ+ρ), $A_{2}=\frac{\frac{\beta }{N}\Lambda (\mu +\rho )}{\mu (\mu +\rho +\alpha )}e^{-\mu \tau }$, $A_{3}=\frac{\delta \frac{\beta }{N}\alpha \Lambda }{\mu (\mu +\rho +\alpha )}e^{-\mu \tau }$, $A_{4}=(\mu +\gamma )$, $A_{5}=(\sigma \lambda +\mu )$.


$$\left|{J_{S_{n}}|}_{D_{1}}-\lambda I\right|=[\begin{array}{cccc} -A_{0}-\lambda & \rho & -A_{2} & 0\\ \alpha & -A_{1}-\lambda & -A_{3} & 0\\ 0 & 0 & A_{2}+A_{3}-A_{4}-\lambda & \sigma \\ 0 & 0 & \gamma & -A_{5}-\lambda \end{array}]=0.$$

$$g_{0}\lambda^{4}+g_{1}\lambda^{3}+g_{2}\lambda^{2}+g_{3}\lambda +g_{4}=0.$$

Where, $g_{0}=1$.


$$g_{1}=\left(A_{0}+A_{1}{-A}_{3}+A_{5}-A_{2}+A_{4}\right).$$

$$\begin{array}{cc} g_{2} & =({A_{0}A}_{1}-{A_{0}A}_{5}-{A_{0}A}_{2}-{A_{0}A}_{3}+{A_{0}A}_{4}+{A_{1}A}_{5}-{A_{1}A}_{2}-{A_{1}A}_{3}\\ & \;+{A_{1}A}_{4}{{-A}_{2}A}_{5}-{A_{3}A}_{5}+{A_{5}A}_{4}+\alpha \rho ). \end{array}$$

$$\begin{array}{cc} g_{3} & =({A_{0}A_{1}A}_{5}-{A_{0}A_{1}A}_{2}-{A_{0}A_{1}A}_{3}+{A_{0}A_{1}A}_{4}-{A_{0}A_{2}A}_{5}-{A_{0}A_{5}A}_{3}+{A_{0}A_{4}A}_{5}\\ & \;-A_{1}A_{2}A_{5}+A_{1}A_{4}A_{5}-A_{1}A_{3}A_{5}+\alpha \rho A_{3}-\alpha \rho A_{2}-\alpha \rho A_{3}+\alpha \rho A_{4}). \end{array}$$

$$g_{4}=\left(A_{1}A_{4}A_{5}-A_{0}A_{1}A_{3}A_{5}-A_{0}A_{1}A_{2}A_{5}+-\alpha \rho A_{2}A_{5}-{\alpha \rho A}_{3}A_{5}+\alpha \rho A_{4}A_{5}+\alpha \rho A_{5}\right).$$

## Appendix B

$P_{0}=\frac{\beta }{N}{I^{*}e}^{-\mu \tau }$, $P_{1}=(\mu +\alpha )$, $P_{2}=\delta \frac{\beta }{N}I^{*}e^{-\mu \tau }$, $P_{3}=(\mu +\rho )$, $P_{4}=\frac{\beta }{N}{S_{u}}^{*}e^{-\mu \tau }$, $P_{5}=\delta \frac{\beta }{N}{S_{v}}^{*}e^{-\mu \tau }$, $P_{6}=(\mu +\gamma )$, $P_{7}=(\sigma \lambda +\mu )$.


$${J_{S_{n}}|}_{D_{2}}=[\begin{array}{cccc} {-P}_{0}-P_{1} & \rho & -P_{4} & 0\\ \alpha & -P_{2}-P_{3} & -P_{5} & 0\\ P_{0} & P_{2} & P_{4}+P_{5}-P_{6} & \sigma \\ 0 & 0 & \gamma & -P_{7} \end{array}].$$

$$\left|{J_{S_{n}}|}_{D_{2}}-\lambda I\right|=\left|\begin{array}{cccc} {-P}_{0}-P_{1}-\lambda & \rho & -P_{4} & 0\\ \alpha & -P_{2}-P_{3}-\lambda & -P_{5} & 0\\ P_{0} & P_{2} & P_{4}+P_{5}-P_{6}-\lambda & \sigma \\ 0 & 0 & \gamma & -P_{7}-\lambda \end{array}\right|=0.$$

$$d_{0}\lambda^{4}+d_{1}\lambda^{3}+d_{2}\lambda^{2}+d_{3}\lambda +d_{4}=0.$$

Where,


$$d_{0}=1.$$

$$d_{1}=\left(P_{0}+P_{1}+P_{2}+P_{3}-P_{4}-P_{5}-P_{6}\right).$$

$$\begin{array}{cc} d_{2} & =(P_{0}P_{7}+P_{1}P_{7}+P_{2}P_{7}+P_{3}P_{7}-P_{4}P_{7}-P_{5}P_{7}-P_{6}P_{7}+P_{0}P_{2}+{P_{0}P}_{3}-P_{0}P_{4}\\ & \;-P_{0}P_{5}+P_{0}P_{6}+P_{1}P_{2}+{P_{1}P}_{3}-{P_{1}P}_{4}-{P_{1}P}_{5}+P_{1}P_{6}+P_{2}P_{4}\\ & \;-P_{2}P_{5}+P_{2}P_{6}{-P_{3}P}_{4}-P_{3}P_{5}+P_{3}P_{6}+P_{2}P_{5}-\rho \alpha +P_{0}P_{4}). \end{array}$$

$$\begin{array}{cc} d_{3} & =(P_{0}P_{2}P_{7}+P_{0}P_{3}P_{7}+P_{0}P_{4}P_{7}-P_{0}P_{5}P_{7}-P_{0}P_{6}P_{7}+P_{1}P_{2}P_{7}+{P_{1}P}_{3}P_{7}-{P_{1}P}_{4}P_{7}\\ & \;-{{P_{7}P}_{1}P}_{5}-P_{1}P_{6}P_{7}-P_{2}P_{4}P_{7}-P_{2}P_{5}P_{7}+P_{2}P_{6}P_{7}{-P_{3}P}_{4}P_{7}-P_{3}P_{5}P_{7}\\ & \;+P_{3}P_{6}P_{7}+P_{2}P_{5}P_{7}-\rho \alpha P_{7}-P_{0}P_{4}P_{7}-P_{0}P_{2}P_{4}-{P_{0}P}_{2}P_{5}\\ & \;+P_{0}P_{2}P_{6}{-P_{0}P_{3}P}_{4}+P_{0}P_{3}P_{5}+P_{0}P_{3}P_{6}+P_{0}P_{2}P_{5}-P_{1}P_{2}P_{4}\\ & \;-P_{1}P_{2}P_{5}+P_{1}P_{2}P_{6}{-P_{1}P_{3}P}_{4}-P_{1}P_{3}P_{5}+P_{1}P_{3}P_{6}+P_{1}P_{2}P_{5}\\ & \;+\rho \alpha P_{5}+\rho \alpha P_{4}-\rho \alpha P_{6}-\rho P_{4}P_{5}+\alpha P_{4}P_{2}+P_{0}P_{2}P_{4}+P_{0}P_{3}P_{4}). \end{array}$$

$$\begin{array}{cc} d_{4} & =({-P}_{0}P_{2}P_{4}P_{7}-{P_{0}P}_{2}P_{5}P_{7}+P_{7}P_{0}P_{2}P_{6}{-P_{0}P_{3}P}_{4}P_{7}-P_{0}P_{3}P_{5}P_{7}+P_{0}P_{3}P_{6}P_{7}\\ & \;+P_{0}P_{2}P_{5}P_{7}-P_{1}P_{2}P_{4}P_{7}-P_{1}P_{2}P_{5}P_{7}+P_{1}P_{2}P_{6}P_{7}{-P_{1}P_{3}P}_{4}P_{7}\\ & \;-P_{1}P_{3}P_{5}P_{7}+P_{1}P_{3}P_{6}P_{7}+P_{1}P_{2}P_{5}P_{7}+\rho \alpha P_{5}P_{7}+\rho \alpha P_{4}P_{7}\\ & \;-\rho \alpha P_{6}P_{7}-\rho P_{0}P_{5}P_{7}+\alpha P_{4}P_{2}P_{7}+P_{0}P_{2}P_{4}P_{7}+P_{0}P_{3}P_{4}P_{7}). \end{array}$$
