# Correction: Dynamical analysis of scabies delayed epidemic model with second-order global stability

**DOI:** 10.1371/journal.pone.0344502

**Published:** 2026-03-06

**Authors:** Emad Fadhal, Ali Raza, Eugénio M. Rocha, Wafa F. Alfwzan, Muhammad Rafiq, Nauman Ahmed, Muhammad Bilal

The Funding statement for this article is incorrect. The correct Funding statement is as follows: Princess Nourah bint Abdulrahman University Researchers Supporting Project No. (PNURSP2025R371), Princess Nourah bint Abdulrahman University, Riyadh, Saudi Arabia. Also, this work was supported by the Deanship of Scientific Research, Vice Presidency for Graduate Studies and Scientific Research, King Faisal University, Saudi Arabia (KFU242952).
